# Co-occurrence of driver and passenger bacteria in human colorectal cancer

**DOI:** 10.1186/1757-4749-6-26

**Published:** 2014-06-25

**Authors:** Jiawei Geng, Qingfang Song, Xiaodan Tang, Xiao Liang, Hong Fan, Hailing Peng, Qiang Guo, Zhigang Zhang

**Affiliations:** 1 Department of Infectious Diseases, The First People’s Hospital of Yunnan Province, Kunming 650032, China; 2 State Key Laboratory of Genetic Resources and Evolution, Laboratory of Evolutionary & Functional Genomics, Kunming Institute of Zoology, Chinese Academy of Sciences, Kunming 650223, China; 3 Department of Gastroenterology, The First People’s Hospital of Yunnan Province, Kunming 650032, China; 4 Faculty of Life Science and Technology, Kunming University of Science and Technology, Kunming 650500, China; 5 Medical Faculty, Kunming University of Science and Technology, Kunming 650500, China

**Keywords:** CRC, Driver bacteria, Passenger bacteria, Co-occurrence

## Abstract

**Background:**

Both genetic and epigenetic alterations have been reported to act as driving forces of tumorigenesis in colorectal cancer (CRC), but a growing body of evidence suggests that intestinal microbiota may be an aetiological factor in the initiation and progression of CRC. Recently, the “driver-passenger” model for CRC has connected these different factors, but little has been done to characterize the CRC gut microbiome.

**Findings:**

Building on the driver-passenger model, we used 454 pyrosequencing of bacterial 16S rRNA genes associated with 10 normal, 10 adenoma, and 8 tumor biopsy samples, and found 7 potential driver bacterial genera and 12 potential passenger bacterial genera (7 being pro-inflammatory and 5 anti-inflammatory). Further analysis also showed certain co-expression patterns among different clusters of bacteria that may potentially be related to the promotion or progression of gut cancers.

**Conclusions:**

The present findings provide preliminary experimental evidence supporting the proposition of bacterial “driver-passenger model” for CRC, and identified potentially novel microbial agents that may be connected to risk of CRC in a Han Chinese population.

## Background

Colorectal cancer (CRC) has long been considered as malignant cell proliferation caused by accumulated genetic and epigenetic mutations [1,2], but increasing evidence suggests that the composition of the human intestinal microbiome may offer novel insights into the aetiology of CRC [3]. If correct, certain intestinal bacterial agents may be significant factors that contribute to the accumulated mutations that often manifest during cancer cell differentiation and development in the gut. From this perspective, Tjalsma *et al.* proposed a bacterial driver-passenger model to explain the involvement of microbial agents in the origin and proliferation of CRC. Under this model, driver and passenger bacteria each play distinct roles in eliciting epithelial phenotype transformation of tissue from normal states, to hyperplasia, and adenoma to carcinoma [4]. Building on this model, we attempted to identify potential driver and passenger bacteria that may be associated with CRC in a Han Chinese population via 454-pyrosequencing analysis of bacterial 16S rRNA genes.

## Methods

We analyzed a total of 28 location-matched biopsy samples, including previously gathered normal (n = 10) [5], and tumor tissues (n = 8) [6], as well as newly sampled adenoma tissues (n = 10), with each sample being taken from one individual subject. All patients and healthy controls were of independent genetic background and of Han Chinese origin, living in Kunming, Yunnan Province, China. Written informed consent was obtained from all participants prior to their inclusion in the study. All protocols and procedures of this study were approved by the Medical Ethics Board of the First People’s Hospital of Yunnan Province of China, and carried out in accordance to all relevant provincial, national and international guidelines.

Normal and tumor tissues were gathered during two previous studies, which each detailed their respective methods [5,6]. For the adenoma samples, following extraction of genomic DNA , the V1-V2 region of the 16S ribosomal RNA (rRNA) gene was amplified via PCR and then subjected to 454 pyrosequencing analysis, as described previously [5,6]. Sequencing reads were quality filtered, OTU clustered (97% sequence identity, equal to bacterial species level), then ChimeraSlayer filtered and further analyzed using the QIIME pipeline [7] and RDP-classifier [8]. OTUs found in ≥20% samples were retained for the further analysis. PLS-DA plotting of samples based on microbiota analysis was performed using METAGENassist, a comprehensive web server software used in comparative metagenomics [9].

Co-occurring network analysis using the Spearman rank correlation was conducted using Hmisc 3.9-3 (Harrell, Vanderbilt University School of Medicine, Nashville, TN, USA) within the R software package, using the relative abundance of different types of bacterial genera. Statistical *P*-values were corrected using the FDR method in the *p*.adjust within the R package. Each co-occurring pair had an absolute Spearman rank correlation above 0.50, with an FDR-corrected significance level under 0.05. The results were transformed into links between two bacterial taxa in the co-occurrence network. Co-occurring networks were visualized using Cytoscape 2.8.2 [10].

All statistical analyses were performed using SigmaPlot 12.0 (Systat Software, Inc.) or relevant programs within the R software package. General characteristics were expressed as mean or median. Multiple samples comparisons were performed using one-way analysis of variance (ANOVA) (parametric) or Kruskal-Wallis one-way ANOVA on ranks (non-parametric).

## Results

After filtering raw data with our set of criteria [6], we obtained a dataset consisting of a total of 100,276 high quality 16S rRNA gene sequences, with an average of 3,581 ± 408 (S.E.) (n = 28) sequences per sample. Within the dataset we identified a total of 767 OTUs, based on 97% sequence similarity (equal to bacterial species level), with an average of 290 ± 16 (n = 28) OTUs per sample. Using the estimation of Good’s Coverage [11,12] showed that 95.20 ± 0.70% of the total found species were represented in any given sample, ensuring completeness and accuracy of data used for further analyses.

**Figure 1:**
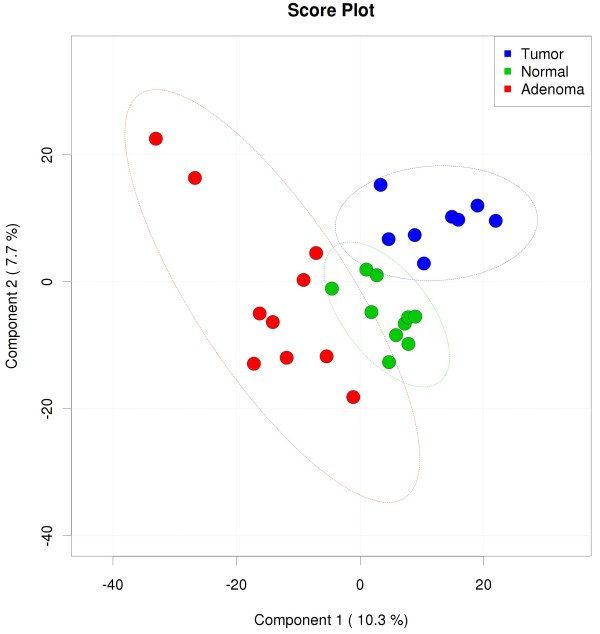
**16S rRNA gene surveys reveal hierarchical partitioning of human normal tissue, adenoma tissue and tumor tissue-associated microbiomes.** Bacterial communities were clustered using partial least squares-discriminant analysis (PLS-DA). Each point corresponds to a sample colored to indicate tumor, adenoma or normal status. The normal biopsy samples are colored by green, the adenomas by red, and the tumors by blue.

**Figure 2:**
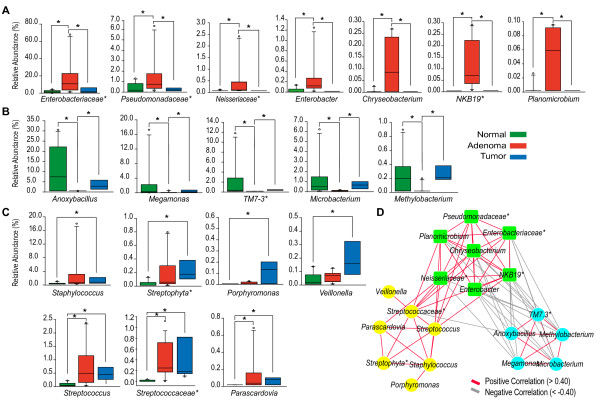
**Identification of potential driver and passenger bacteria associated with CRC and their co-occurrences.** Unclassified taxa were represented by star. ^*^FDR-corrected *P* < 0.05. **(A)** Potential driver bacteria whose relative abundances in adenoma tissues were significantly higher than in normal and tumor tissues; **(B)** Potential anti-inflammatory passenger bacteria whose relative abundances in adenoma tissues were significantly lower than in normal and tumor tissues; **(C)** Potential pro-inflammatory passenger bacteria whose relative abundances in normal tissues were significantly lower than in tumor or/and adenoma tissues; **(D)** Correlation relationships among three types of CRC bacteria as described **(A)**-**(C)**. Each co-occurring pair has an absolute Spearman rank correlation above 0.50 with an FDR-corrected significance level under 0.05. Node labels are corresponding to bacterial taxa. Edges are colored by positive or negative correlations. Driver bacterial cluster was colored by green, anti-inflammatory passenger bacterial cluster by blue and pro-inflammatory passenger bacterial cluster by yellow.

PLS-DA analysis illustrated a distinct structural segregation for all 28 samples that appears to be primarily related to health/disease conditions rather than other factors (*e.g.*, inter-individual differences) (Figure 1). The driver-passenger model proposed by Tjalsma [4] holds that CRC-associated bacterial drivers can be defined as intestinal bacteria with pro­carcinogenic features that may potentially initiate CRC development, while bacterial passengers are gut bacteria known to exist within the gut microbiome of patients with advanced-CRC, which should have a competitive advantage in the tumor microenvironment, allowing them to outcompete bacterial drivers of CRC. Using these definitions, we identified 7 bacterial genera as potential driver bacteria (Figure 2A) and 12 bacterial genera as potential passenger bacteria (Figure 2B and C). Since the identified passenger bacteria may influence either the suppression or promotion of tumor development [4], we further examine these bacteria and identified 7 of the 12 passenger bacterial genera as potential pro-inflammatory agents with low abundance in normal tissue (Figure 2C), and the remaining 5 genera as potential anti-inflammatory agents with high abundance in both normal and tumor tissues (Figure 2B).

Presuming that bacteria play functional roles in the progression of CRC, then within the CRC microbiome, bacteria with specific functions should be either co-existed or co-occurred. Correlation analysis of the 19 bacterial taxa (described in Figure 2A-C) showed that bacterial taxa with the same defined role were clustered into groups with positive correlation of each other (Figure 2D). These positive associations partially support the “driver and passenger bacteria” notion. We also found that the driver bacterial cluster was significantly and positively correlated to the pro-inflammatory passenger bacterial cluster (Figure 2D), suggesting that the presence of driver bacteria may drive the colonization of tumor-foraging opportunistic pathogens (*e.g.*, *Streptococcus* spp.). Conversely, the anti-inflammatory passenger bacterial cluster was significantly and negatively correlated with the driver bacterial cluster (Figure 2D), implying that anti-inflammatory passenger bacteria may primarily be restricted to the early phases of carcinogenesis.

## Discussion

Collectively, our results suggest a potentially dynamic and previously unknown interaction among intestinal mucosal bacteria that may markedly impact the occurrence or suppression of tumor development within the gut. Consistent with the earlier findings of Tjalsma et al. [4], our analysis identified members of *Enterobacteriaceae* (Figure 2A) as potential bacterial drivers, and *Streptococcaceae* (Figure 2C) as possible pro-inflammatory passenger bacteria. This finding was not unexpected, since earlier reports considered both *Escherichia coli* (*Enterobacteriaceae*) and *Streptococcus gallolyticus* (*Streptococcaceae*) as protagonists of tumor development due to the correlation of their presence and increased risks of CRC [3]. Despite these general similarities, there were some marked differences among our studied Han Chinese population. Previously, the gut enterotoxigenic *Bacteroides fragilis* and *Fusobacterium* spp. were respectively found to act as driver bacteria and pro-inflammatory passenger bacteria [3,4] but in our present study, neither appeared in significant abundance differences across any of the sampled tissues. Heterogeneity across the gut microbiome between different populations may explain this difference, with those two bacterial taxa being potentially and weakly linked to CRC among a Chinese population.

The differences between our studied population and those found in previous is not unexpected, as numerous reports have found evidence suggesting that the CRC gut microbiomes vary considerably by population, age or biogeographic position. For instance, there are significant regional variations in CRC microbiota, with the well-known *Fusobacterium* spp. being more abundant in colon tumors from Spanish populations as compared to those in the United States or Vietnam [13]. There are also age-dependent divergences in the CRC microbiomes of younger and older patients [14]. Earlier studies also ignored variations in microbiome composition based sample locations, which can vary considerably, even among patients. For example, there are significant differences in microbial structure and community composition between normal fecal and mucosal samples [15], especially among CRC patients [16]. Similar variations are also found between samples obtained at different positions along the normal intestinal tract [5]. Differences in analysis, methodology (*e.g.*, phylogeny, culturing, and metagenomics), and sample size can also lead to markedly different findings [17].

Aside from the observed similarities and differences between our study and the previous reports [13,16,18-22], we also identified several new potential driver bacteria (*e.g.*, unclassified *Pseudomonadaceae* and *Neissenaceae*) (Figure 2A) and pro-inflammatory passenger bacteria (*e.g.*, S*taphylococcus* and *Veillonella*) (Figure 2C). Previous reports found that *Pseudomonadaceae* were markedly increased in the stools of patients with end-stage renal disease [23], while *Staphylococcus* was considerably related to advanced-stage colon cancer [24] and *Veillonella* to gut disorders among patients with minimal hepatic encephalopathy [25]. Together, these findings suggest that the novel bacterial agents we identified in this study may be related to CRC progression. Further study of these novel genera may help fully elucidate their function within the gut microbiome, as well as their potential associations with CRC. Curiously, we also noted a discrepancy in the distribution of certain anti-inflammatory bacteria (Figure 2B) enriched in normal intestinal mucosa [5] but decreased in CRC patients [26]. This discrepancy suggests that the anti-inflammatory bacteria we observed may function in some manner that delays the progression of CRC, potentially by preserving intestinal niches or producing compounds that exhibit anti-carcinogenic activities [4]. Unfortunately, the precise effect of these bacteria on the tumor microenvironment remains unclear, but such anti-inflammatory bacteria may prove viable targets for researches into gut disorders or CRC therapeutics.

Taken on the whole, our results—especially those which differ from those in previous reports—serve as the reminders of the difficulty inherent in examining the relationship between gut disorders, gut microbiome composition and structure, and larger genetic or environmental factors. One advantage of the driver-passenger model is that it combines several of these factors into a more comprehensive framework that helps explain the etiology and underlying mechanisms behind gut disorders like CRC. However, the observed heterogeneity of the gut microbiome due to different populations, disease status, or sample locations highlights the need for alternative approaches that can more adequately characterize changes to the microbiome that often accompany—or potentially underlie—gut disorders like CRC. More effective models, such as the driver-passenger model, may be able to better explain the correlations between changes in the gut microbiome composition and structure, thereby leading to improved diagnostics.

## Conclusion

In conclusion, our results largely support the proposition of the bacterial driver-passenger model for CRC proposed by Tjalsma et al. [4]. The potential driver and passenger bacteria we identified in the present study also offer further evidence into exploring the relationship between changes in the gut microbiome composition and structure and CRC. Further replication with a larger sample size will likely help develop a more generally applicable pattern of CRC microbiome variations in Chinese populations, and provide foundational evidence needed to fully elucidate the observed heterogeneity between different populations with CRC.

## Abbreviations

CRC: Colorectal cancer; OTUs: Operational taxonomic units; rRNA: Ribosomal RNA.

## Competing interests

The authors declare that they have no competing interests.

## Authors’ contributions

JG, QS and ZZ performed research, analyzed data, and wrote the manuscript; XL, XT, HF, and HP performed research; QG and ZZ conceived the study and commented on the manuscript. All authors read and approved the final manuscript.

## Authors’ information

JG, XL: Department of Infectious Diseases, The First People’s Hospital of Yunnan Province, Kunming, China. XT, HF, QG: Department of Gastroenterology, The First People’s Hospital of Yunnan Province, Kunming, China. QS: Faculty of Life Science and Technology, Kunming University of Science and Technology, Kunming, China. HP: Medical Faculty, Kunming University of Science and Technology, Kunming, China. ZZ: State Key Laboratory of Genetic Resources and Evolution, Kunming Institute of Zoology, Chinese Academy of Sciences, Kunming, China

## Data sharing

454 sequence data obained from 10 adenoma biopsy samples in this study was deposited to the NCBI Sequence Read Archive (SRA) (accession number SRP041558) (http://www.ncbi.nlm.nih.gov/sra).
